# ‘I’ve Changed My Mind’, Mindfulness-Based Childbirth and Parenting (MBCP) for pregnant women with a high level of fear of childbirth and their partners: study protocol of the quasi-experimental controlled trial

**DOI:** 10.1186/s12888-016-1070-8

**Published:** 2016-11-07

**Authors:** Irena K. Veringa, Esther I. de Bruin, Nancy Bardacke, Larissa G. Duncan, Francisca J. A. van Steensel, Carmen D. Dirksen, Susan M. Bögels

**Affiliations:** 1 Research Institute Child Development and Education (RICDE), Faculty of Social and Behavioral Sciences, Research Priority Area Yield, University of Amsterdam, Nieuwe Achtergracht 127, 1018 WS Amsterdam, The Netherlands; 2 UvA minds, academic outpatient child and adolescent treatment center of the University of Amsterdam, Plantage Muidergracht 14, 1018 TV Amsterdam, The Netherlands; 3 Osher Center for Integrative Medicine and Department of Nurse-Midwifery, University of California-San Francisco (UCSF), UCSF box 1726, San Francisco, CA 94143-1726 USA; 4 Department of Human Development and Family Studies and Department of Family Medicine and Community Health, University of Wisconsin-Madison, Madison, WI 53706 USA; 5 Clinical Epidemiology and Medical Technology Assessment (KEMTA), Maastricht University Medical Center, P. Debyelaan 25, 6202 AZ Maastricht, The Netherlands

**Keywords:** Fear of childbirth, Labour pain, Mindfulness, Obstetrical interventions, Cost-effectiveness

## Abstract

**Background:**

Approximately 25 % of pregnant women suffer from a high level of Fear of Childbirth (FoC), as assessed by the Wijma Delivery Expectancy Questionnaire (W-DEQ-A, score ≥66). FoC negatively affects pregnant women’s mental health and adaptation to the perinatal period. Mindfulness-Based Childbirth and Parenting (MBCP) seems to be potentially effective in decreasing pregnancy-related anxiety and stress. We propose a theoretical model of Avoidance and Participation in Pregnancy, Birth and the Postpartum Period in order to explore FoC and to evaluate the underlying mechanisms of change of MBCP.

**Methods/Design:**

The ‘I’ve Changed My Mind’ study is a quasi-experimental controlled trial among 128 pregnant women (week 16–26) with a high level of FoC, and their partners. Women will be allocated to MBCP (intervention group) or to Fear of Childbirth Consultation (FoCC; comparison group). Primary outcomes are FoC, labour pain, and willingness to accept obstetrical interventions. Secondary outcomes are anxiety, depression, general stress, parental stress, quality of life, sleep quality, fatigue, satisfaction with childbirth, birth outcome, breastfeeding self-efficacy and cost-effectiveness. The total study duration for women is six months with four assessment waves: pre- and post-intervention, following the birth and closing the maternity leave period.

**Discussion:**

Given the high prevalence and severe negative impact of FoC this study can be of major importance if statistically and clinically meaningful benefits are found. Among the strengths of this study are the clinical-based experimental design, the extensive cognitive-emotional and behavioural measurements in pregnant women and their partners during the entire perinatal period, and the representativeness of study sample as well as generalizability of the study’s results. The complex and innovative measurements of FoC in this study are an important strength in clinical research on FoC not only in pregnant women but also in their partners.

**Trial registration:**

Dutch Trial Register (NTR): NTR4302, registration date the 3rd of December 2013.

## Background

Fear of childbirth (FoC) is a highly prevalent negative emotion among pregnant women characterized by high levels of stress and emotional maladaptation to the normal physiological and psychological processes of being pregnant and giving birth [1, 2]. Reports demonstrate that approximately 25 % of pregnant women suffer from a high level of FoC, as assessed by the Wijma Delivery Expectancy/Experience Questionnaire (W-DEQ-A and B), defined as a W-DEQ-A score ≥66 [3]. Besides, approximately 10 % of pregnant women have been found to suffer from severe FoC (W-DEQ-A score ≥85) [4]. The complex causes of FoC can be examined from a biopsychosocial perspective [5] that includes biologically-oriented dimensions of fear (e.g., fear of pain, fear of bodily harm, or fear of one’s own or one’s infant’s death), psychological factors (e.g., personality traits, a history of traumatic life events or previous difficult or traumatic obstetrical experiences, feelings of helplessness, or anxiety about motherhood), and social factors (e.g., dissatisfaction with the partner relationship, lack of social support, low socioeconomic status, hearing ‘horror stories’ about labour from family, friends, acquaintances, and media sources) [1, 6, 7]. FoC can be categorized as ‘primary FoC’ occurring in nulliparous women (first-time mothers) and ‘secondary FoC’ following a previous difficult or traumatic childbirth experience. Differences in severity of FoC between nulliparous and parous women are still being investigated [8, 9]. FoC seems to be a specific domain of anxiety associated with, yet distinct from, general anxiety or depression [10]. Only a small number of studies have evaluated the content and interrelationship of pregnant women’s FoC and their partners’ FoC [11, 12]; the role of fathers’ perinatal distress as a contributing factor to FoC among pregnant women is still unknown.

### Consequences of FoC

Studies have shown that FoC negatively affects women in a number of ways, including sleep disturbance and depression in pregnancy [13], increased health care use during the perinatal period [14], requests for medical interventions such as an elective caesarean section, *a priori* request of epidural analgesia without pain experience [15–18], negative experience of childbirth, postpartum depression, post-birth trauma [19–21], and low rates of breastfeeding [22, 23]. Negative effects of FoC are also associated with increased incidence of small gestational age (15 %), increased preterm birth rate (12 %), infant admission to intensive care [24, 25] as well as poor quality of infant’s sleep [26].

### Increasing FoC in labouring women in the Netherlands?

The prevalence of FoC in the Dutch population of pregnant women as assessed by the W-DEQ-A is unknown. However, 47 % of first time Dutch mothers do report fear of childbirth [27]. Maladaptation during childbirth in Dutch women can be seen in the increasing numbers of non-urgent medical referrals during labour [28]. The Dutch midwifery-led model of care assumes that pregnancy, birth and the postnatal period are healthy life events for a mother and her baby. This care is offered in independent midwifery practices in the community and in hospitals. If or when complications arise, women are referred to obstetrician-led care and new-borns are referred to paediatric care.

The most recent data, collected in 2000–2008, evaluating the Dutch midwifery-led care system showed that while almost 84 % of all pregnant women started prenatal care in primary midwifery-led care, only 29 % of them actually gave birth under the supervision of a midwife. This means that 71 % of all births took place in secondary obstetrician-led care settings. In 2014 this trend remained stable [29]. Overall, almost 60 % of the medical referrals were for non-urgent conditions, such as the need for pain relief, augmentation of labour with oxytocin, or instrumental deliveries due to prolonged labour. However, these referrals did not lead to better child outcomes (such as fewer new-borns with a five-minute Apgar score below 7 or a lower rate of natal or neonatal mortality) when compared with births in primary care [28].

### Management of FoC in midwifery-led care in the Netherlands

Currently, the most commonly applied strategy to prevent and guide FoC in the perinatal period in midwifery practice in The Netherlands is for pregnant women to attend antenatal classes and to write a birth plan. However, studies of individuals and groups in antenatal education have questioned the efficacy of these programmes in preparing expectant couples for the challenges of childbirth and early parenting. A large body of research on structured educational programmes provided during pregnancy and offered in midwifery care reported no consistent results of the effects on knowledge acquisition, antenatal anxiety, maternal sense of control, labour pain, use of medication, psychological adjustment to parenthood and obstetrical interventions [30, 31]. Birth plans take into account the preferences of the pregnant woman and her partner regarding medical management of the childbirth experience [32]. One of the main purposes of these birth plans, which were developed in the 1980’s in many other Western countries, was to increase a woman’s feelings of control over her birthing situation, as well as to reduce the medicalization of childbirth. Studies assessing the effects of using birth plans showed a small improvement in dealing with fear, pain and the overall childbirth experience [33, 34].

These findings may suggest that the management of FoC should include more specific cognitive strategies to identify and shift patterns of cognition that may be potentially distressing to women in labour [35].

### Studies evaluating psychological strategies in the management of FoC

Randomized controlled trials (RCT’s) and prospective cohort’s studies reporting positive effects of interventions including psychological strategies such as psychoeducation, cognitive-behavioural therapy or mindfulness-based programmes (MBP’s) in pregnant women with FoC are limited. In Table 1 a summary of the currently available studies is provided. As can be seen, two large randomized trials with an active control group [36, 37] and one non-randomized trial [38] demonstrated small to moderate effects of psychoeducational based programmes on the reduction of FoC, while one small pre- and post-study evaluating cognitive behavioural therapy on FoC reduction in pregnant women with sever FoC showed a large effect size [39]. Another four studies reported large effects of MBP’s on the reduction of FoC or pregnancy related anxiety in different populations of pregnant women [40–43]. However, the sample sizes of these studies were small and only two of them were randomized controlled trials [42, 43].

**Table 1:** Overview of Studies on Psychological Interventions for Reduction of FoC and Pregnancy-related Anxiety

Research group	Research design, (*n*), population	Type of experimental intervention	Primary outcome post intervention
Duncan & Bardacke (2010) [40] United States of America.	Uncontrolled pre- and post-study. (*n* = 27). Community sample of pregnant women.	Nine 3-hours group sessions of MBCP led by a midwife -mindfulness instructor.	Reduced anxiety (PAS) with a large effect *within* the EI group: Cohen’s *d* = 0.81, *p* < 0.0001
Fontein et al. (2016) [38] The Netherlands.	Non-randomized trial. (*n* = 433). Pregnant women with maternal distress.	*WazzUp Mama*?! internet-delivered programme supported by midwives vs. CAU.	EI > CAU reduced anxiety (PRAQ) with a moderate effect *between* the groups: Cohen’s *d* = 0.64, *p* < 0.001
Goodman et al. (2014) [41] United States of America.	Uncontrolled pre- and post-study. (*n* = 23). Pregnant women with anxiety symptoms.	Eight 2-hours group sessions of CALM led by a mindfulness instructor.	Reduced anxiety (BAI) with a large effect *within* the EI group: Cohen’s *d* = 0.83, *p* < 0.001
Guardino et al. (2014) [42] United States of America.	Randomized controlled trial. (*n* = 47). Pregnant women with stress.	Six 2-hours group sessions of MAPS led by a mindfulness instructor vs. CAU.	EI > CAU reduced anxiety (PSA) with a large effect *between* the groups: Cohen’s *d* = 0.77, *p* < 0.05
Nieminen et al. (2016) [39] Sweden.	Prospective cohort study. (*n* = 28). Nulliparous pregnant women with sever FoC.	Eight weeks ICBT programme supported by a therapist.	Reduced FoC (W-DEQ-A) with a large effect *within* the EI group: Cohen’s *d* = 0.95, *p* < 0.0005
Rouhe et al. (2012) [36] Sweden.	Randomized controlled trial. (*n* = 371). Pregnant women with a sever FoC.	Six 2-hours session of psychoeducational group therapy led by psychologists vs. CAU.	EI > CAU reduced FoC (W-DEQ-B) with a small effect *between* the groups: Cohen’s *d* = 0.35, *p* = 0.02
Thoohill et al. (2014) [37] Australie.	Randomized controlled trial. (*n* = 198). Pregnant women reporting FoC.	Two psychoeducational telephone sessions led by trained midwives vs. CAU.	EI > CAU reduced FoC (W-DEQ-A) with a moderate effect *between* the groups: Cohen’s *d* = 0.59, *p* < 0.001
Vieten & Astin (2008) [43] United States of America.	Randomized controlled waitlist trial. (*n* = 31). Pregnant women with mood concerns.	Eight 2-hours group sessions of Mindful Motherhood led by a psychologist-mindfulness instructor vs. CAU.	EI > CAU reduced anxiety (STAI) with a large effect *between* the groups: Cohen’s *d* = 0.85, *p* < 0.04

*Note: BAI* Back Anxiety Inventory, *CALM* Coping with Anxiety through Living Mindfully, *CAU* care-as-usual, *EI* experimental intervention, *ICBT* Internet-delivered Cognitive Behavioral Therapy, *MAPS* Mindful Awareness Practice Sessions, *MBCP* Mindfulness-Based Childbirth and Parenting, *PAS* Pregnancy Anxiety Scale, *PRAQ* Pregnancy Related Anxiety Questionnaire, *PSA* Pregnancy Specific Anxiety, *STAI* State-Trait Anxiety Inventory, *W-DEQ-A* Wijma Delivery Expectancy Questionnaire-version A, *W-DEQ-B* Wijma Delivery Expectancy Questionnaire-version B

Given the limited amount of evidence-based effective psychological interventions for reduction of FoC in pregnant women, and the relatively unknown effects of the existent interventions on the birth-related outcomes more large randomized controlled trials in this field should be recommended. Further, as the effect sizes of the MBP’s appear to be higher than those of other psychological interventions (see Table 1), high quality RCT’s comparing MBP’s to control treatments are in need.

### A theoretical model of avoidance and participation in pregnancy, birth and the postpartum period

**Fig. 1:**
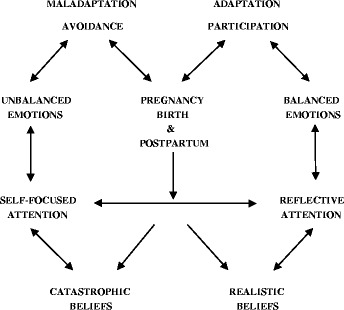
A Theoretical Model of Avoidance and Participation in Pregnancy, Birth and the Postpartum Period

Figure 1 shows a theoretical model of Avoidance and Participation in Pregnancy, Birth and the Postpartum Period. This model can serve as a heuristic in which current psychological knowledge and research on the effects of beliefs and emotions on women’s behaviour in pregnancy, birth and the postpartum period is integrated. We adapted elements of Vlaeyen’s Fear-Avoidance Model of Pain [44], Beck’s Cognitive Theory [45], and Lazarus and Folkman’s Stress and Coping Theory [46] in this model. Two opposite behavioural responses to pregnancy, birth and the postpartum period are postulated, namely avoidance and participation. Attention is the point of engagement for change in the presented cognitive-emotional pathways and behaviours (see Fig. 1).

In accordance with Cognitive Theory, a belief is a state of mind in which a person thinks something to be true with or without empirical evidence. An individual’s beliefs are the result of cognitive-emotional information processing starting at an early age of perception. Beliefs guide individuals’ behavioural (i.e., approach or avoidance) and psychophysiological responses (i.e., arousal). Cognitive-emotional information processing can be biased by self-focused attention to a certain event, which may lead to biased core beliefs about the event [45]. Biased core beliefs such as catastrophic beliefs (irrational worst-case outcomes) lead to maladaptation. Maladaptation is a trait that is or has become more harmful than helpful. Its source can be related to a personal experience (i.e., trauma, environment), education (i.e., lack of knowledge), and biological predisposition (i.e., genetics). Clinical studies have shown a potentially causal role of catastrophic beliefs in developing unbalanced emotions, such as anxiety, fear and depression [47]. However, the relation between beliefs, attention, emotions, and behaviour is likely to be bidirectional. In accordance with Stress and Coping Theory [46] a person with unbalanced emotions will avoid the stressful event and appraise the event as too overwhelming to adapt to. Adaptation is a process of change by which a person becomes better suited to an event. Avoidance, a maladaptive type of behaviour, may reduce distress in the short term, but will maintain and strengthen the unbalanced emotions, since by avoiding the event the catastrophic beliefs are not disconfirmed and realistic beliefs are not generated.

How can these theories be applied to the perinatal situation? Pregnant women with catastrophic beliefs view the perinatal period in terms of danger and harm that may occur in the future: during pregnancy (e.g., *‘My baby will die’),* birth (e.g., ‘*Labour pain will predominate everything’*) and the postpartum period (e.g., ‘*My recovery will take too long’*). They have a hyper focus on danger (self-focused attention) rather than appraising these perinatal events in terms of relevance and reality. Perinatal catastrophic beliefs can contribute to pregnant women’s behavioural (e.g., avoidance or participation), emotional (e.g., fear, stress, depressed mood) and psychophysiological (e.g., higher levels of stress hormones) maladaptation to the natural process of being pregnant, birthing, postpartum recovery and mothering [9, 17, 18, 22]. It can therefore be expected that pregnant women with catastrophic beliefs and unbalanced emotions will attempt to exempt themselves from any effort to approach to distressful perinatal events (e.g., by requesting *a priori* epidural anaesthesia or an elective caesarean section). A negative spiral may be the result; avoidance may further strengthen unbalanced emotions, leading to postpartum depression or posttraumatic stress syndrome and future maladaptive behaviours, such as avoidance of pregnancy, natural birth, contact with the baby and social contacts [1, 48].

The opposite of catastrophic beliefs are realistic beliefs about pregnancy, birth and the postpartum period, the counter half of the model. Realistic beliefs are characterized by a reflective attention for causes and conditions of the perinatal events due to unbiased cognitive-emotional information processing. In this half of the model, relationships between appraisals, attention, and emotions can also be considered bidirectional. Therefore, it can be expected that pregnant women with realistic perinatal beliefs and balanced emotions will participate (approach), instead of avoid, an event (e.g., giving birth) due to positive appraisal of the reality and their ability to adapt to the perinatal events.

Since the quality of attention can influence the cognitive-emotional- information processing and behaviours in pregnant women, interventions targeting the quality of attention and developing adaptive behaviours towards perinatal events are in need of more psychological midwifery research.

### Mindfulness-based programmes and their mechanisms of action

MBP’s such as Mindfulness-Based Stress Reduction (MBSR) [49] and MBCT [50] have become widely used in health care settings and have shown to be effective for a variety of psychological and physical conditions including depression, anxiety, stress [51], and chronic pain [52] in both clinical and non-clinical populations. A more recent application of mindfulness is mindful parenting. Recent data suggest that mindful parenting effectively reduces parental stress, parental psychopathology, child psychopathology, and improves parenting and co-parenting [53, 54].

These MBP’s are based on Buddhist meditation practices. Mindfulness can be defined as “the awareness that arises from paying attention, on purpose, in the present moment, and non-judgmentally” [49]. The non-judgmental quality of the attention during meditation practice allows individuals to observe physical sensations, thoughts, and emotions, to work at accepting them as they are, and thereby reduce automatic reactions to them [55]. Mindfulness practice helps the practitioner realize that physical sensations, thoughts and emotions are continuously changing. In addition, with on-going practice of mindfulness meditation, feelings of caring and kindness toward oneself and others, and compassion for the common human experience may arise [56]. The above-mentioned skills seem to be key processes of change underlying the MBP’s positive outcome.

Whether MBP’s also improve the well-being of parents-to-be is, as of yet, largely unknown. There are early indications that MBP’s reduce perinatal anxiety, depression and the severity of labour pain in various populations of pregnant women [40–43, 57]. A good example is the well-developed Mindfulness-Based Childbirth and Parenting (MBCP) programme. MBCP, as evaluated in a pilot study (*n* = 27), seems to be potentially effective considering the significant large effect size in the decrease in pregnancy-related anxiety (Cohen’s *d* = 0.81), the increase in non-reactivity (Cohen’s *d* = 0.85), and increase in positive affect (Cohen’s *d* = 0.40) found among pregnant women who participated in the MBCP program [40]. MBCP is a childbirth education program that integrates mindfulness meditation with current knowledge of the neurobiological processes of the perinatal period.

We hypothesize three underlying mechanisms of action of the effectiveness of MBCP. First, an increase in mindful awareness, which is defined as the ability to observe moment to moment internal and external experiences in body and mind, to describe these experiences, to respond rather than react towards inner experiences or events, and to be more accepting. Second, an increase in self-compassion, meaning being moved by one’s own suffering, experiencing kindness towards inherent shortcomings, and acknowledging one’s own experience being a part of the common human experience. And third, a decrease in catastrophic beliefs, such as worries about anticipated events and experiences.

Given the promising impact of MBP’s on fear and stress, and the promising application for women experiencing pregnancy-related anxiety, in this study we will evaluate the effectiveness of MBCP on FoC in a population of pregnant women with a high level of FoC during the perinatal period. We will use a quasi-experimental design to compare MBCP with a structured version of care- as-usual: Fear of Childbirth Consultations (FoCC).

### Aims

The ‘I’ve Changed My Mind study’ is designed with four primary aims: 1) to assess the effects of MBCP, as compared to FoCC, on the primary outcome measures of a) FoC, b) labour pain, and c) willingness to accept obstetrical interventions without medical indications in pregnant women with a high level of FoC; 2) to assess the effects of MBCP, as compared to FoCC, on the secondary outcome measures of a) anxiety, b) depression, c) general stress, d) pre- and postnatal stress, e) quality of life, f) sleep quality of women, her partner and infant, g) pre- and postnatal fatigue, h) satisfaction with childbirth, i) birth outcome for mother and child, and j) breastfeeding self-efficacy in pregnant women with a high level of FoC and their partners; 3) to examine overall mindful awareness, self-compassion, and catastrophic beliefs as possible mediating mechanisms underlying the effectiveness of MBCP; 4) to assess the costs of health care use due to FoC and cost-effectiveness of MBCP as compared to FoCC. These four aims will be examined in three time periods: a) during pregnancy, b) after labour, and c) during the maternity leave period following the birth.

It is hypothesized that participants in the MBCP group, as compared to those in the FoCC group will a) show larger and longer lasting effects on all primary and secondary outcome measures, b) demonstrate increased overall mindful awareness and self-compassion, and decreased catastrophic beliefs, and c) have lower FoC related health care costs, indicating that MBCP is more cost-effective than FoCC.

## Methods

### Design

The study design is a quasi-experimental controlled trial with two arms (intervention and active comparison group) involving four assessment time points. Participants will be allocated by the order of inclusion in the study (alternation). Inclusion will take place at baseline 16–26 weeks gestational age. Subsequently, questionnaires will be filled in one-two weeks pre-intervention (T1) and post-intervention (T2), and two-four weeks (T3) and 16–20 weeks (T4) following the birth. Participating couples will receive a fee of €50 upon completion of data collection (T4).

**Fig. 2:**
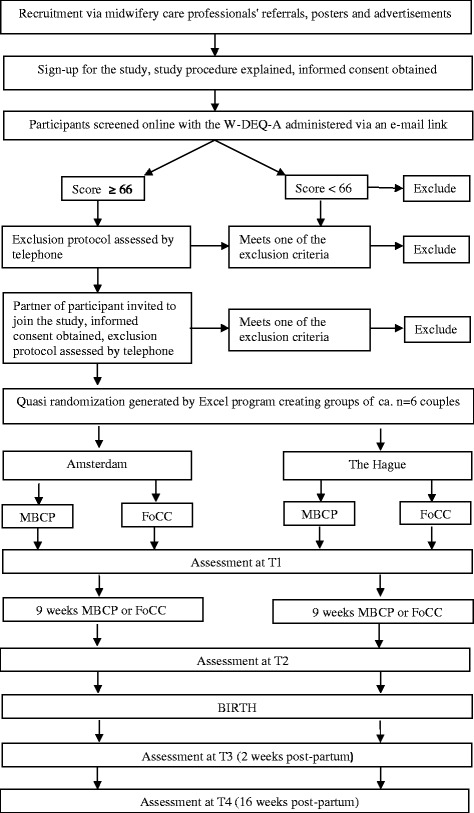
Flow-chart of Inclusion of the ‘I’ve Change My Mind Study’

Participants from an urban area in The Netherlands will be quasi-randomized to the intervention (MBCP) or comparison group (FoCC) using an Excel program of created codes with the intent of producing equivalent training group cohorts of approximately six participants each. The flowchart of the study design and participants is depicted in Fig. 2.

### Sample size

Assuming a medium effect size of MBCP compared to the comparison condition and referrals of 30 % from primary to secondary midwifery care due to maladaptation to childbirth [28] we aim to include *n* =128 pregnant women with a high level of FoC and their partners (64 couples in each arm) to achieve a power of 80 % to find a significant effect (test of between-within interaction, 5 % alpha, 0.5 correlation). We based our power calculations on a medium effect size versus the large effect size found in a single-group trial of MBCP for decreasing pregnancy anxiety (*d* = 0.81, *p* < 0.0001) in the study of Duncan and Bardacke [40] and the overall medium mean effect size of MBP’s (*d* = 0.59) [55], in part due to the unknown effect size of the FoCC comparison condition.

### Participants

This trial will be conducted in primary and secondary midwifery care settings in Amsterdam and The Hague, The Netherlands. All study procedures and informed consent forms received approval from the Ethics Review Board of the Faculty of Social and Behavioural Sciences at the University of Amsterdam (certificate number 2013-CDE-3064). This trial is registered in the Dutch Trial Register (NTR) under number 4302. Participation is entirely voluntary and pregnant women and/or their partners can stop participating at any time without having to sign anything or provide a reason for stopping.

### Inclusion criteria

Participants are primiparous and multiparous women, aged ≥18, fluent in the Dutch or English language who are more than 16 weeks and less than 26 weeks pregnant at baseline, and are experiencing a high level of FoC (W-DEQ-A ≥66) [9]. If an eligible woman agrees to participate, her partner will be asked to take part in the study as well. As ‘partner’ we refer to the father or co-parent of the expected baby, or a significant other person related to the pregnant woman who will be present at the birth. Pregnant women may enter the study without a partner or a significant other.

### Exclusion criteria

Exclusion criteria for this study are: (a) previous acute psychotic episode or diagnosed psychotic disorder; (b) current suicidal risk; (c) current substance use and dependency; (d) borderline personality disorder in the pregnant woman or her partner; (e) current trauma unrelated to childbirth traumatic stress disorder; or (f) participation in a MBP within the past year. The use of antidepressant medication, as long as the prescribed dose remains stable during the study, participation in an on-going psychological intervention or a prenatal education course, or a childbirth trauma as assessed by the Traumatic Event Scale (TES-B) [58] are not exclusion criteria. Women with a multiple gestation, HIV infection, or at high risk for premature labour will be excluded. Please see Procedure section below for a description of a two-stage exclusion protocol that will be employed.

### Intervention: The Mindfulness-Based Childbirth and Parenting (MBCP) programme

The MBCP programme [40] developed in the United States by midwife and mindfulness teacher Nancy Bardacke, CNM, MA, is a formal adaptation of MBSR specifically developed for the expectant parent population. The teachings of mindfulness through formal and informal meditations are fully integrated with the knowledge of the psychobiological processes in pregnancy, birth, breastfeeding, postpartum adjustment and the psychobiological needs of the infant. MBCP as developed by Bardacke includes nine weekly three-hour classes, a 7-hour day of silent meditation practice and a 3.5-hour reunion gathering after all the babies have been born. For purposes of this study, the standard MBCP was adapted (with permission by Bardacke). Two first classes were combined as Class 1 and the 7-hour day of silent meditation practice was reduced to one 3-hour class (see Table 2). Expectant parents are asked to commit to practicing the formal meditations at home for 30 min a day, six days a week with instructional CDs that are given as part of the course materials. Adherence to the intervention is assessed by reporting the number of classes attended and weekly diaries of the amount of time spent between sessions in formal daily meditation practice and being mindful of the activities of daily living (informal practices). The importance of keeping a meditation diary will be emphasized. Each MBCP course will be taught by an experienced midwife, who, in accordance with the MBCP protocol, has been trained as an MBCP teacher by Nancy Bardacke. All MBCP sessions will be video recorded to assure fidelity to the MBCP model. Ratings of fidelity to the MBCP model will be carried out by two independent raters. The MBCP sessions will be free of charge and take place at a Mindfulness Centre in Amsterdam and The Hague.

**Table 2:** Outline of the Dutch Adaptation of the Mindfulness-Based Childbirth and Parenting Programme

Week 1	Background of mindfulness, the MBCP programme, introduction to mindfulness meditation through an eating meditation, awareness of breathing meditation.
Week 2	Body scan meditation, attitudinal foundations of mindfulness, community building.
Week 3	Awareness of breathing meditation, body scan meditation, psycho-education: physiology of childbirth from a body-mind perspective.
Week 4-6	Yoga, sitting meditation, pain meditations using ice and a variety of pain-coping strategies, expanding the capacity to “be with” unpleasant/challenging sensations in the body and unpleasant or stressful thoughts and emotions, 3-Minute Breathing Space meditation, exploration of the notion of “being in control” during childbirth, psycho-education: baby's journey through the pelvis.
Week 7	Session of silence, body scan, yoga, sitting meditation, mindful eating, walking meditation, mindful speaking and listening inquiry practice regarding fears and joys around childbirth and the life change the couple is living.
Week 8	Loving-kindness meditation, psychoeducation: biological, emotional and social needs of the newborn and mindfulness practice for moment to moment caretaking, the needs of the postpartum family.
Week 9	Psychoeducation: physiology of breastfeeding, mindfulness as a skill for coping with breastfeeding challenges and postpartum adjustment, closing ceremony.

### Active Comparison Condition: Fear of Childbirth Consultation (FoCC)

The target sample of pregnant women included in this study suffer from intense FoC. In order to acknowledge the fears of these women we have upgraded care as usual into structured consultations on FoC (FoCC). FoCC consists of an adaptation of the Biopsychosocial Model [5] and the Childbirth Plan of the Royal Dutch Organization of Midwives (KNOV) [59].

This individualized programme for expectant couples includes two consultation sessions with a trained midwife of one-hour each over a nine-week period (see Table 3). The aim of FoCC is to gain insight into the variety of specific factors playing a role in the origin and presence of fear and stress around pregnancy, birth and the postpartum period as well as designing a suitable coping plan based on the particular fears and stresses, and includes some components of psychoeducation about fear. A structured form is used to collect information about FoC related factors and for the coping plan. The coping plan may include referral to a psychologist or other mental health care services. All FoCC sessions will be audio recorded in order to assure treatment fidelity. Two recordings per consultation will be randomly selected and evaluated by an independent midwife. The free of charge FoCC sessions will take place at the participant’s midwifery practice or place of residence.

**Table 3:** Outline of the Two Sessions of the Fear of Childbirth Consultations

FoCC First Consultation
Mapping of the bodily, mental and social factors underlying FoC and the postpartum period. Interview about the pregnant woman’s overall state of physical health and current pregnancy, her mental health and emotional state, her ideas and values regarding being pregnant, the process of giving birth and being a parent, incidence of psychopathology in her family of origin, her most severe fears about childbirth and the postpartum period, her relationship with her partner, family, presence of social support, workplace experiences, important life events and potential vulnerabilities. A written psychoeducation about FoC and matching behavior from a body and mind perspective is provided.
FoCC Second Consultation
Designing an Individual Childbirth Plan based on the findings from the first consultation and the pregnant woman’s wishes, including the care provider’s, partner’s and family’s attitudes towards her upcoming childbirth, the woman’s intrinsic coping strategies regarding childbirth including her approach to labour pain and 2nd stage pushing, potential requests for care and guidance from her care provider and family, her ability to adapt to possible medical interventions, and guidance regarding first contact with her newborn.

### Outcome measures

In this study several assessment tools will be used. Some measures were translated specifically for this study. Translations were made in accordance with the scientific standards for translating questionnaires [60] and permission to translate has been given by the original authors. Table 4 presents an overview of the primary and secondary outcome measures and the time points of study assessments.

**Table 4:** Overview of Measures, Outcomes, and Corresponding Measurement Occasions for the (pregnant) Women and their Partners

Measure	Outcome domain	Measurement occasion			
		T1	T2	T3	T4
W-DEQ-A	Anticipated fear of childbirth	X	X		
W-DEQ-B	Experienced fear of childbirth			X	X
PDSS^a^	Perinatal disaster scenarios	X	X	X	X
PAFS	Responses to anxiety and fear	X	X	X	X
CLP	Catastrophizing of labour pain	X	X		
LPAQ	Acceptance of labour pain	X	X		
VAS	Labour pain intensity	X	X	X	
WAOI^a^	Willingness to accept interventions	X	X		
DASS-21^a^	Depression, anxiety and stress	X	X	X	X
PSS^a^	Stress	X	X	X	X
EPDS	Prenatal/postnatal depression	X	X	X	X
PES-US	Uplifting experience of pregnancy	X	X		
NPSI-SF^a^	Parenting stress			X	X
EQ-5D^a^	Quality of life	X	X	X	X
PSQI^a^	Sleep quality	X	X	X	X
HSDQ-I^a^	Insomnia	X	X	X	X
ISVIS^a^	Interpretation of infant sleep	X	X	X	X
BISQ	Infant sleep			X	X
MAF^a^	Fatigue	X	X	X	X
SIL^a^	Satisfaction with birth			X	X
MR	Medical report about perinatal period				X
BSES-SF	Breastfeeding self-efficacy			X	X
FFMQ^a^	Mindful awareness	X	X	X	X
IM-P^a^	Mindful parenting skills			X	X
SCS-SF^a^	Self-compassion	X	X	X	X
PHCQ	Costs in and outside the healthcare sector	X	X	X	X

*Note*. ^a^instruments are filled in by (pregnant) woman and partner, others are filled in by (pregnant) women only. T1 = pre-intervention; T2 = post-intervention; T3 = two -four weeks after birth; T4 = 16–20 weeks after birth. MBCP or FoCC takes place between T1 and T2. *BISQ* Brief Infant Sleep Questionnaire, *BSES-SF* Breastfeeding Self-Efficacy Scale-Short Form, *CLP* Catastrophizing Labour Pain, *DASS-21* Depression Anxiety Stress Scale, *EPDS* Edinburgh Prenatal/Postnatal Depression Scale, *EQ-5D* Five-Dimensional EuroQol, *FFMQ* Five Facets Mindfulness Questionnaire, *HSDQ-I* Holland Sleep Disorders Questionnaire-Insomnia subscale, *IM-P* Interpersonal Mindfulness in Parenting scale, *ISVIS* Infant Sleep Vignettes Interpretation Scale, *LPAQ* Labour Pain Acceptance Questionnaire, *MAF* Multidimensional Assessment of Fatigue, *MR* Medical Report, *NPSI-SF* Nijmeegse Parental Stress Index-Short Form, *PAFS* Perinatal Anxiety/Fear Scale, *PES-US* Pregnancy Experience Scale-Uplifts Subscale, *PDSS* Perinatal Disaster Scenarios Scale, *PHCQ* Perinatal Healthcare Costs Questionnaire, *PSQI* Pittsburgh Sleep Quality Index, *PSS* Perceived Stress Scale, *SCS-SF* Self Compassion Scale Short-Form, *SIL* Salomon’s Item List, *WAOI* Willingness to Accept Obstetrical Interventions, *W-DEQ-A* Wijma Delivery Expectancy Questionnaire-version A, *W-DEQ-B* Wijma Delivery Expectancy Questionnaire-version B

### Primary outcome measures

Primary outcome measures are (a) FoC, (b) labour pain, and (c) willingness to accept obstetrical interventions without medical indications.

The complexity of FoC will be measured by three instruments, namely the W-DEQ- A and B [3], the newly developed Perinatal Disaster Scenarios Scale (PDSS), (Veringa IK, van Berge S, Wouters A, de Bruin EI: PDSS, unpublished), and the experimental Perinatal Anxiety/Fear Scale (PAFS), (Veringa IK, de Bruin EI, Bögels S: PAFS, unpublished). First, anticipated and experienced levels of FoC will be assessed with the 33 items self-report W-DEQ- A and B covering several domains of FoC: (a) general fear, (b) negative appraisal, (c) loneliness, (d) lack of self-efficacy, (e) lack of positive anticipation, and (f) concerns about the child [61]. Second, the individual perinatal fear-eliciting beliefs, in pregnant women and their partners, will be assessed by the PDSS. We developed the PDSS, which is based on the Social Phobia Belief Scale (SPBS) [62], for this study in order to describe catastrophic beliefs about childbirth and future-related events that are eliciting fear (maximum of 3 beliefs). The PDSS assesses the probability of actual occurrence of those catastrophic events, the severity, and the ability to cope with them in the future on the Visual Analogue Scale (VAS) [63], (0–100 %). And third, to assess responses to anxiety and fear in pregnant women we will administer the experimental PAFS based on the Dimensional Anxiety Self-report of Social Phobia level 3 DSM-IV [64].

Subsequently, three instruments will be included to assess labour pain. First, anticipated and experienced cognitive and emotional components of labour pain will be assessed by the 13 item self-report Catastrophizing Labour Pain (CLP) subscale derived from the Labour Pain Cognitions and Coping List (LPCCL) [65], the 20 item self-report Labour Pain Acceptance Questionnaire (LPAQ), (Veringa IK, Wouters A, Lowe S, Langedijk L, de Bruin E: LPAQ, unpublished), an adaptation of the Chronic Pain Acceptance Questionnaire (CPAQ) [66], and the expected and experienced severity of labour pain will be assessed by the VAS [63] (0–10).

Last, the willingness to accept obstetrical interventions without medical indications will be assessed by the Dutch version of the Willingness to Accept Obstetrical Interventions measure (WAIO) [67], (Veringa IK, Wouters A, Lowe S, de Bruin EI: Dutch version of WAIO, unpublished).

### Secondary outcome measures

Secondary outcome measures are a) anxiety, b) depression, c) general stress, d) stress, e) quality of life, f) sleep quality, g) fatigue, h) satisfaction with childbirth, i) birth outcome for mother and infant, and j) breastfeeding self-efficacy.

Anxiety, depression, and general stress will be assessed by the Depression, Anxiety and Stress Scale (DASS-21) [68]. Psychological stress, the degree to which individuals appraise events in their lives as stressful, will be assessed by the Perceived Stress Scale (PSS) [69]. In addition, current perinatal depression symptoms will be assessed by using the Edinburgh Prenatal/Postnatal Depression Scale (EPDS) [70]. Pregnancy stress will be assessed by the Dutch version of the Pregnancy Experience Scale (van der Zwan JE, de Vente W, Koot HM, Huizink AC: Validation of the Dutch version of the Pregnancy Experience Scale for pregnant women and partners of pregnant women, under review) using the uplifts subscale (PES-US) derived from the Pregnancy Experience Scale (PES) [71]. Subsequently, parental stress after birth will be assessed by the Nijmeegse Parental Stress Index-Short Form (NPSI-SF) [72]. Quality of life will be assessed by the Five-Dimensional EuroQol instrument (EQ-5D) [73], which assesses mobility, self-care, usual activities, pain/discomfort and anxiety/depression.

Sleep quality will be assessed by the Pittsburgh Sleep Quality Index (PSQI) [74] with additional sleep efficiency questions, and the Insomnia scale derived from the Holland Sleep Disorders Questionnaire (HSDQ-I) [75]. Sleep quality of the infant will be measured by the Dutch version of the Infant Sleep Vignettes Interpretation Scale (ISVIS) [76], (van Berge S, Veringa IK, Wouters A, de Bruin EI: Dutch version of ISVIS, unpublished) [76], and by the Brief Infant Sleep Questionnaire (BISQ) [77]. Furthermore, fatigue will be assessed by the Multidimensional Assessment of Fatigue (MAF) [78]. Satisfaction with childbirth will be assessed by the Dutch version of the Salomon’s Item List (SIL) [79]﻿, (﻿﻿﻿Veringa IK, Wouters A, Lowe S, de Bruin EI: Dutch version of SIL, unpublished﻿﻿). Birth outcome for mother (e.g., modus partus) and infant (e.g., birth weight and APGAR score) will be derived from the medical report (MR). And last, breastfeeding self-efficacy of the mother will be assessed by the Dutch version of the Breastfeeding Self-Efficacy Scale-Short Form (BSES-SF) [80]﻿, (Veringa IK, Wouters A, Lowe S, de Buin EI: Dutch version of BSES-SF, unpublished﻿).

### Mechanisms of action and process evaluation

Changes in overall mindful awareness, self-compassion, and catastrophic beliefs are hypothesised to be potential underlying working mechanisms of MBCP leading to positive changes in mental health and behaviour during the perinatal period. Overall mindful awareness including qualities such as observing, describing, acting with awareness, non-judging and non-reactivity to inner experience, will be assessed by the Five Facet Mindfulness Questionnaire (FFMQ) [81]. In addition, mindful awareness specifically related to one’s role as a (new) parent will be assessed with the Interpersonal Mindfulness in Parenting Scale (IM-P) [82]. Self-compassion will be assessed by the Self-Compassion Scale-Short Form (SCS- SF) [83], and catastrophic beliefs will be assessed by the Perinatal Disaster Scenarios Scale (PDSS).

Expectancy effects in women and partners will be assessed by the question: “If you had a choice, which one of the study’s programmes would you prefer to participate in?”. Adherence to MBCP will be assessed by number of classes attended and weekly diaries of the number of minutes spent in formal meditation practice each week between sessions. Data regarding the number of attended sessions of FoCC will also be collected.

### Cost-effectiveness evaluation

The evaluation of cost-effectiveness will be carried out from a societal and health care perspective including direct and indirect costs, with an average time frame of six months following study inclusion. Participants will fill out a standardized Perinatal Health Care-costs Questionnaire (PHCQ) in which they are retrospectively asked how often they had contact with the health care system, including type, duration, medications used, number of days absent from work, production losses, and professional and family support. At T1participants are asked to report any contacts with the healthcare system from the time of first knowledge of pregnancy to the start of MBCP or FoCC - which contains information about the past three to five months; from T1 till T4 participants report about the past three months. Costs of both programmes will be calculated separately based on the duration and frequency of sessions and group size. Costs will be derived by multiplying the resources used by the unit price of each resource. Unit prices will be based on Dutch standard prices from the Dutch Guideline of Cost Research [84] or other published unit prices. The costs of the interventions will be based on the standardized hourly pay of midwives and the invested intervention related educational costs. The EQ-5D [73] is administrated to provide utilities and to calculate quality adjusted life years (QALY’s).

### Recruitment

Figure 2 provides an overview of the recruitment and study procedures. Recruitment for this study started in April 2014. Midwives and obstetricians were briefed on the study at workshops and fraternity meetings. Pregnant women are invited to join the study in two ways: via advertisement posters and brochures in midwifery waiting rooms inviting them to visit the study’s website http://www.mbcpmidwife.nl/ or by midwives and obstetricians who find they are caring for a highly anxious and/or stressed pregnant woman and offer them information about the study. After a potentially eligible pregnant woman or her care provider contacts the research team, informed consent is obtained. Subsequently, the pregnant woman completes an online screening questionnaire (W-DEQ-A) [3]. Questionnaire responses are scored within 48 h. Women who score ≥66 on the W-DEQ-A are contacted by telephone and the study’s two-stage exclusion protocol is administered. After this procedure, the eligible participant’s partner is invited to join the study, informed consent is obtained and the two-step exclusion interview is administered.

The first step of the exclusion protocol is carried out by the research midwife by telephone in order to identify a current risk for a psychosis/psychotic disorder, potential suicidal risk, substance abuse and dependency, or borderline personality disorder in the woman and/or her partner. In cases where any of the above risks or disorders are suspected, an extensive personal psychological interview is conducted by a trained psychologist using the Structured Clinical Interview for DSM-IV disorders Axis I and Axis II (SCID-I and SCID-II) [64]. Subsequently the general physician of the participant is informed of the existing or risk for a particular mental disorder.

Recruitment will continue until at least 64 participants with a W-DEQ-A score ≥66 have completed the study’s programme in each of two study arms.

### Quasi-experimental allocation to the two study conditions

The allocation to the two study groups will be done on the sequence of entry into the study using an Excel program of created codes. This procedure ensures that the referral midwives and obstetricians are not able to predict the group to which the participant will be assigned. The choice for quasi-experimental allocation is based on a steadily increasing gestational age, dependence on recruitment speed and efficiency, and the required minimum group size of six participants in the MBCP intervention group.

### Statistical analyses

#### Primary analyses

Dichotomous outcome data will be analysed using Chi-square tests or logistic regression using the method of ‘last observation carried forward’ (i.e., assuming no change) to handle dichotomous incomplete data. Continuous outcome data will be analysed with Multi-level analyses. Multi-level analyses with full information maximum likelihood (FIML) estimation use all available data and allow intention-to-treat analyses including all participants with incomplete data and participants who dropped out during the study. Continuous variables will be transformed into *Z*-scores. In this way, the parameter estimates can be interpreted as a measure of effect: i.e., as Cohen’s *d* for dichotomous predictors and as *r* for continuous predictors. Outliers will be identified. Analyses will be run twice: once in which all original scores will be included and once in which outliers will be changed to *Z*-scores (-) 3.29. Dependent variables will be level of FoC, labour pain and willingness to accept obstetrical interventions without medical indications. Predictors will be the different time measurements (T2, T3, and T4 against T1) and condition (MBCP versus FoCC). Interaction effects of time X condition will be added to the model to examine which programme is more effective over time.

#### Secondary analyses

Multilevel mediation analyses will be conducted to evaluate the possible underlying mechanisms of action in MBCP. In these analyses only participants considered to be “treated” i.e., those who have received at least five out of nine MBCP sessions will be included. We will examine the mediating effect of general mindful awareness, self-compassion, and catastrophic beliefs.

### Cost-effectiveness

Incremental costs effectiveness ratio (ICER) will be calculated and expressed as (a) the cost per woman that displays a significantly reduced level of FoC, and (b) the cost per QALY. Standard sensitivity analyses will be performed to test for the robustness of the cost-effectiveness result. Non-parametric bootstrapping method will be used, performing 1000 replications of the original costs data, to produce confidence intervals around the costs estimates and quantify uncertainty around the calculated ICERs [85]. Cost-effectiveness planes will be used to represent the bootstrapped ICERs: the horizontal line reflects the difference in effect and the vertical line reflects the difference in costs. Cost-effectiveness acceptability curves will be used to inform decision-makers on the probability that the studied intervention is cost-effective at a range of ceiling ratios.

### Dissemination recommendation

In order to inform future dissemination of MBCP into midwifery care we will evaluate the value of MBCP for midwifery care taking into account clinical relevance. Two pillars will provide perspective on clinical relevance: (I) the clinical significance of the effects and (II) acceptability and feasibility of MBCP for the participants. To assess the clinical significance of MBCP, we will compare the pre- and post-treatment raw scores of primary and secondary measures with current established norms relevant for a population of pregnant women. To assess acceptability and feasibility of MBCP, participants will be asked to complete an evaluation form about the personal value they found from participating in the program, along with our assessment of the number of sessions attended and adherence to home practice.

## Discussion

This will be the first RCT comparing the effects of MBCP to FoCC on an array of childbirth and early parenting outcomes in pregnant women with a high level of FoC and their partners. This study will provide greater insight into the psychological processes underlying the occurrence, development and responses to FoC. Given the high prevalence and severe negative impact of FoC for pregnant women and their infants, this study can be of major importance if statistically and clinically meaningful benefits are found. Addressing the problem of FoC is critical and the proposed study evaluates an innovative MBCP that holds the potential of being an effective, non-invasive, and non-medical intervention for pregnant women with FoC, whit the potential for widespread dissemination that builds on the popularity of MBP’s. Further, we expect a potentially stronger effect of MBCP than FoCC on adaptation to the perinatal period, and a decrease in not-urgent medical interventions during childbirth. A reduction in unnecessary medical interventions has the potential to reduce or redirect the costs of midwifery care towards a more preventive approach for women and their partners in the perinatal period.

Some limitations to this study design need to be considered. First, the quasi-experimental study design creates less homogeneous groups then with full randomization (RCT) and permits a greater risk of bias due to potential alternation and allocation problems. To work with this, we adopted the recommendations for designing the Q-RCT studies from the Cochrane Handbook for Systematic Reviews of Interventions [86]. Due to the steadily increasing gestational age, dependency on recruitment speed and efficiency, and the required size of approximately six participants in the intervention group, we are obliged to use the quasi-randomization procedure in order to be able to conduct this study efficiently. A second limitation is the uncertainty of the power of this study due to the unknown effect size of the comparison group. To cover this limitation, we have chosen to downsize the expected large effect size of MBCP as shown in Duncan and Bardacke’s [40] study to a medium effect size in order to not under-power our study. An additional limitation is the potentially sizable dropout of participants due to their possibly strong preferences for one of the study’s interventions, the nine weeks duration of the programmes, and the relatively long and direct follow up after the birth. As a retention strategy we provide the participants with a financial incentive (€50) for completing the measurements following the birth.

Among the strengths of this study are the clinical-based experimental design, the extensive cognitive-emotional and behavioural measurements in pregnant women and their partners during the entire perinatal period, and the representativeness of the study sample as well as the generalizability of the study’s results. The complex and innovative measurements of FoC in this study are an important strength in research on FoC not only in pregnant women but also in their partners.

In the future, it would be interesting to evaluate the effects of MBCP on the physiological pathways of the stress response, such as the hypothalamic–pituitary–adrenal (HPA) axis, and maternal and foetal levels of corticosteroids in relation to perinatal outcomes for mother and baby. A study of the implementation and dissemination of MBCP into midwifery practice in the Netherlands would be the next logical step in MBCP research. Future research on the effects of mindful parenting on the mother-infant relationship, assessments of infant emotional expression and regulation, and stress due to fear, anxiety or depression in new mothers will allow for continuity between research and treatment for women at risk.

With this study we also aim to increase awareness among maternity caregivers of the important effects of maternal psychological wellbeing during the processes of adaptation to pregnancy, childbirth and parenting. Findings from this study would help midwives in their role of signalling, referring, cooperating with psychologists and preparing expectant women and their partners who are experiencing consequences of FoC in pregnancy, during childbirth and in parenting. Midwives are an ideal group of professionals to incorporate MBCP into their midwifery practices in The Netherlands for prevention and co- treatment purposes, because of the frequent and intimate contact with pregnant women and new families.

## Abbreviations

BISQ: Brief Infant Sleep Questionnaire
BSES-SF: Breastfeeding Self-Efficacy Scale-Short Form
CLP: Catastrophizing labour pain
CPAQ: Chronic pain acceptance questionnaire
DASS-21: Depression, anxiety, stress scale
DSM-IV: Diagnostic and statistical manual of mental disorders – 4th edition
EPDS: Edinburgh Prenatal/Postnatal Depression Scale
EQ-5D: EuroQol – 5 Dimensional instrument
FFMQ: Five facet mindfulness questionnaire
FoC: Fear of childbirth
FoCC: Fear of childbirth consultation
HSDQ: Holland sleep disorders questionnaire
ICER: Incremental costs effectiveness ratio
IM-P: Interpersonal Mindfulness in Parenting Scale
ISVIS: Infant Sleep Vignettes Interpretation Scale
KNOV: Koninklijke Nederlandse Organisatie van Verloskundigen
LPAQ: Labour pain acceptance questionnaire
MAF: Multidimensional Assessment of Fatigue
MBCP: Mindfulness-Based Childbirth and Parenting
MBCT: Mindfulness-based cognitive therapy
MBIs: Mindfulness-based interventions
MBSR: Mindfulness-Based Stress Reduction
MR: Medical report
NCCIH: National Centre for Complementary
NPSI-SF: Nijmeegse Parental Stress Index-Short Form
NTR: Nederlands Trial Register
PAFS: Perinatal Anxiety/Fear Scale
PDSS: Perinatal Disaster Scenarios Scale
PE: Program evaluation
PES: Pregnancy Experience Scale
PHCQ: Perinatal health costs questionnaire
PP: Program preferences
PSQI: Pittsburgh Sleep Quality Index
PSS: Perceived Stress Scale
QALY’s: Quality adjusted life years
Q-RCT: Quasi randomized controlled trial
RCT: Randomized controlled trial
SCID: Structured Clinical Interview DSM Disorders
SCS-SF: Self-compassion scale-short form
SDE: Social demographic economic characteristics
SIL: Salomon’s Item List
SPBS: Social phobia belief scale
SSRI’S: Selective Serotonin Reuptake Inhibitors
TES-B: Traumatic Event Scale
USNIH: United States National Institutes of Health
VAS: Visual analogue scale
WAOI: Willingness to Accept Obstetrical Interventions measure
W-DEQ-A: Wijma Delivery Expectancy Questionnaire-version A
W-DEQ-B: Wijma Delivery Experience Questionnaire-version B

## References

[1] Hofberg K, Ward M (2004). Fear of childbirth, tocophobia, and mental health in mothers: the obstetric psychiatric interface. Clin Obst Gyneco.

[2] Saisto T, Halmesmäki E (2003). Fear of childbirth: a neglected dilemma. Acta Obstet Gynecol Scand.

[3] Wijma K, Alehagen S, Wijma B (2002). Development of the delivery fear scale. J Psychosom Obstet Gynaecol.

[4] Kjaergaard H, Wijma K, Dykes A-K, Alehagen S (2008). Fear of childbirth in obstetrically low-risk nulliparous women in Sweden and Denmark. J Reprod Infant Psychol.

[5] Engel G (1977). The need for a New medical model: a challenge for biomedicine. Science.

[6] Zar M, Wijma K, Wijma B (2001). Pre- and postpartum fear of childbirth in nulliparous and parous women. Scand J Behav Ther.

[7] Saistoa T, Salmela-Arob K, Nurmic J, Halmesma ÈE (2001). Psychosocial characteristics of women and their partners fearing vaginal childbirth. Br J Obstet Gynaecol.

[8] Alehagen S, Wijma K, Wijma B (2000). Fear during labor. Acta Obstet Gynecol Scand.

[9] Ryding EL, Wijma B, Wijma K, Rydhström H (1998). Fear of childbirth during pregnancy may increase the risk of emergency ceaserean section. Acta Obstet Gynecol Scand.

[10] Zar M, Wijma K, Wijma B (2001). Relations between anxiety disorders and fear of childbirth during late pregnancy. Clin Psychol Psychother.

[11] Hildingsson I (2014). Swedish couples’ attitudes towards birth, childbirth fear and birth preferences and relation to mode of birth - a longitudinal cohort study. Sex Reprod Healthc.

[12] Eriksson C, Westman G, Hamberg K (2006). Content of childbirth-related fear in Swedish women and men-analysis of an open-ended question. J Midwifery Womens Health.

[13] Hall W, Hauck Y, Carty E, Hutton E, Fenwick J, Stoll K (2009). Childbirth fear, anxiety, fatigue, and sleep deprivation in pregnant women. J Obstet Gynecol Neonatal Nurs.

[14] Andersson L, Sundstrom-Poromaa I, Wulff M, Astrom M, Bixo M (2004). Implications of antenatal depression and anxiety for obstetric outcome. Obstet Gynecol.

[15] Waldenström U, Hildingsson I, Ryding EL (2006). Antenatal fear of childbirth and its association with subsequent caesarean section and experience of childbirth. BJOG.

[16] Nieminen K, Stephansson O, Ryding EL (2009). Women’s fear of childbirth and preference for caesarean section a cross-sectional study at various stages of pregnancy in Sweden. Acta Obstet Gynecol Scand.

[17] Alehagen S, Wijma B, Lundberg U, Wijma K (2005). Fear, pain and stress hormones during childbirth. J Psychosom Obstet Gynaecol.

[18] Van den Bussche E, Crombez G, Eccleston C, Sullivan MJ (2007). Why women prefer epidural analgesia during childbirth: the role of beliefs about epidural analgesia and pain catastrophizing. Eur J Pain.

[19] Soderquist J, Wijma B, Thorbert G, Wijma K (2009). Risk factors in pregnancy for post-traumatic stress and depression after childbirth. BJOG.

[20] Robertson E, Grace S, Wallington T, Stewart DE (2004). Antenatal risk factors for postpartum depression: a synthesis of recent literature. Gen Hosp Psychiatry.

[21] Alder JL, Breitinger G, Granado C, Fornaro I, Bitzer J, Hösli I, Urech C (2011). Antenatal psychobiological predictors of psychological response to childbirth. J Am Psychiatr Nurses Assoc.

[22] Ferber SG, Feldman R (2005). Delivery pain and the development of mother infant interaction. J Inter Soc Infant Stud.

[23] O’Keane V, Marsh MS (2007). Depression during pregnancy. BMJ.

[24] Class QA, Lichtenstein P, Långström N, D’Onofrio BM (2011). Timing of prenatal maternal exposure to severe life events and adverse pregnancy outcomes: a population study of 2.6 million pregnancies. Psychosom Med.

[25] Loomans EM, van Dijk AE, Vrijkotte TGM, van Eijsden M, Stronks K, Gemke RJBJ, van den Bergh BRH (2013). Psychosocial stress during pregnancy is related to adverse birth outcomes. Eur J Public Health.

[26] Tikotzky L, Sadeh A (2009). Maternal sleep-related cognitions and infant sleep: a longitudinal study from pregnancy through the 1st year. Child Dev.

[27] DELIVER (2011). Primary midwifery care database.

[28] Offerhaus P, Hukkelhoven C, de Jonge A, van der Pal-de Bruin K, Largo-Jansen A (2013). Persisting rise in referrals during labour in primary midwife-led care in the Netherlands. Birth.

[29] Perinatal Registration in the Netherlands. Utrecht: Stichting Perinatale Registratie Nederland, 2013.

[30] Gagnon AJ, Sandall J (2007). Individual or group antenatal education for childbirth or parenthood, or both. Cochrane Database Syst Rev.

[31] Bergström M, Kieler H, Waldenström U (2009). Effects of natural childbirth preparation versus standard antenatal education on epidural rates, experience of childbirth and parental stress in mothers and fathers: a randomised controlled multicentre trial. BJOG.

[32] Inch S (1998). Birth plans and protocols. J Royal Soc Med.

[33] Lundgren I, Berg M, Lindmark G (2003). Is the childbirth experience improved by a birth plan?. J Mid Womans Health.

[34] Berg M, Lundgren I, Lindmark G (2003). Childbirh experience in women at high risk: is It improved by use of a birth plan?. J Perintal Educ.

[35] Escott D, Slade P, Spiby H (2009). Preparation for pain management during childbirth: The psychological aspects of coping strategy development in antenatal education. Clin Psychol Rev.

[36] Rouhe H, Salmela-Aro K, Toivanen R, Tokola M, Halmesmaki E, Ryding E, Saisto T (2015). Group psychoeducation with relaxation for severe fear of childbirth improves maternal adjustment and childbirth experience – a randomised controlled trial. J Psychosom Obstet Gynaecol.

[37] Toohill J, Fenwick J, Gamble J, Creedy DK, Buist A, Turkstra E, Ryding E (2014). A randomized controlled trial of a psycho-education intervention by midwives in reducing childbirth fear in pregnant women. Birth.

[38] Fontein YJ, Ausems M, De Vries R, Nieuwenhuijze MJ (2016). The effect of *Wazzup Mama?!* An antenatal intervention to prevent or reduce maternal distress in pregnancy. Arch Womens Ment Health.

[39] Nieminen K, Andersson G, Wijma B, Ryding E, Wijma K (2016). Treatment of nulliparous women with severe fear of childbirth via the Internet: a feasibility study. J Psychosom Obstet Gynaecol.

[40] Duncan LG, Bardacke N (2010). Mindfulness-based childbirth and parenting education: promoting family mindfulness during the perinatal period. J Child Fam Stud.

[41] Goodman JH, Guarino A, Chenausky K, Klein L, Prager J, Petersen R, Forget A, Freeman M (2014). CALM Pregnancy: results of a pilot study of mindfulness-based cognitive therapy for perinatal anxiety. Arch Womens Ment Health.

[42] Guardino CM, Dunkel Schetter C, Bower JE, Lu M, Smalley SL (2014). Randomised controlled pilot trial of mindfulness training for stress reduction during pregnancy. Psychol Health.

[43] Vieten C, Astin J (2008). Effects of mindfulness-based intervention during pregnancy on prenatal stress and mood: results of a pilot study. Arch Womens Ment Health.

[44] Vlayean JWS, Linton SJ (2000). Fear-avoidance and its consequences in chronic musculoskeletal pain: a state of the art. Pain.

[45] Beck AT (1976). Cognitive therapy and the emotional disorders.

[46] Lazarus RS, Folkman S (1984). Stress, appraisal and coping.

[47] Alloy LB, Abramson LY, Whitehouse WG, Hogen ME, Panzarella C, Rose DT (2006). Prospective incidence of first onsets and recurrences of depression in individuals at high and low cognitive risk for depression. J Abnorm Psychol.

[48] Flink IK, Mroczek MZ, Sullivan MJL, Linton SJ (2009). Pain in childbirth and postpartum recovery – The role of catastrophizing. Eur J Pain.

[49] Kabat-Zinn J (2005). Coming to our sense.

[50] Segal ZV, Williams JMG, Teasdale JD (2002). Mindfulness-based cognitive therapy for depression: A new approach to preventing relapse.

[51] Khoury B, Lacomte T, Fortuin G, Masse M, Therien P, Bouchard V, Chapleau M, Paquin K, Hofman S (2013). Mindfulness-based therapy: A comprehensive meta-analysis. Clin Psychol Rev.

[52] Reiner K, Tibi L, Lipsitz J (2013). Do mindfulness-based interventions reduce pain intensity? a critical review of the literature. Pain Med.

[53] Bögels SM, Hellemans J, van Deursen S, Römer M, van der Meulen R (2014). Mindful parenting in mental health care: effects on parental and child psychopathology, parental stress, parenting, coparenting, and marital functioning. Mindfulness.

[54] Singh NN, Lancioni GE, Winton ASW, Karazsia BT, Myers RE, Latham LL, Singh J (2014). Mindfulness-based positive behavior support (MBPBS) for mothers of adolescents with autism spectrum disorder: effects on Adolescents’ behavior and parental stress. Mindfulness.

[55] Baer RA (2003). Mindfulness training as a clinical intervention: A conceptual and empirical review. Clin Psychol Sci Prac.

[56] Keng S, Smoski MJ, Robins CJ, Ekblad AE, Brantly JG (2012). Mechanisms of change in mindfulness-based stress reductiona: self-compassion and mindfulness as mediators of interventions outcomes. J Cogn Psyhother.

[57] Duncan LG, Cohn M, Chao M, Cook J, Riccobono J, Bardacke N (2014). Mind in labor: effects of mind/body training on childbirth appraisals and pain medication use during labor. J Altern Complement Med.

[58] Wijma K, Soderquist J, Wijma B (1997). Posttraumatic stress disorder after childbirth: a cross sectional study. J Anxiety Disord.

[59] KNOV Geboorteplan: http://www.knov.nl/vakkennis-en-wetenschap/tekstpagina/253/geboorteplan/.

[60] Beaton DE, Bombardier C, Guillemin F, Bosi FM (2000). Guidelines for the process of cross-cultural adaptation of self-report measures. SPINE.

[61] Garthus-Niegel S, Størksen HT, Torgersen L, Von Soest T, Eberhard-Gran M (2011). The wijma delivery expectancy/experience questionnaire: a factor analytic study. J Psychosom Obstet Gynaecol.

[62] Voncken M, Bögels S (2006). Changing interpretation and judgmental bias in social phobia: a pilot study of a short, highly structured cognitive treatment. J of Cog Psych.

[63] McCormack HM, de Horne DJL, Sheather S (1998). Clinical applications of visual analogue scales: a critical review. Psych Med.

[64] First MB, Gibbon M, Spritzer RL, Williams JBW (1996). User guide for the structured clinical interview for DSM-IV axis 1 disorders.

[65] Veringa IK, Buitendijk S, de Miranda E, de Wolf S, Spinhoven P (2011). Pain cognitions as predictors of the request for pain relief during the first stage of labor: a prospective study. J Psychosom Obstet Gynaecol.

[66] McCracken LM, Vowles KE, Eccleston C (2004). Acceptance of chronic pain: Component analysis and a revised assessment method. Pain.

[67] Green JM, Baston HA (2007). Have women become more willing to accept obstetric interventions and does this relate to mode of birth? Data from a prospective study. Birth.

[68] Lovibond SH, Lovibond PF (1995). Manual for the depression anxiety stress scales.

[69] Cohen S, Williamson G, Spacapan S, Oskamp S (1988). Perceived stress in a probability sample of the United States. The social psychology of health.

[70] Cox JL, Holden JM, Sagovsky R (1987). Detection of postnatal depression: development of the 10-item Edinburgh postnatal depression scale. Brit J Psychiatry.

[71] Dipietro JA, Christensen AL, Costigan KA (2008). The pregnancy experience scale-brief version. J Psychosom Obstet Gynaecol.

[72] Brock A, de Vermulst AA, Gerris JRM, Abidin RR (1992). Nijmeegse Ouderlijke Stress Index. Handleiding experimentele versie.

[73] The EuroQol (1990). EuroQol a new facility for the measure of health related quality of life. Health Policy.

[74] Buysse DJ, Reynolds CF, Monk TH, Berman SR, Kupfer DJ (1989). The Pittsburgh Sleep Quality Index (PSQI): A new instrument for psychiatric research and practice. Psychiatry Res.

[75] Kerkof GA, Geuke ME, Brouwer A, Rijsman RM, Schimsheimer RJ, Van Kasteel V (2013). Holland sleep disorders questionnaire: a new sleep disorders questionnaire based on the international classification of sleep disorders. J Sleep Res.

[76] Sadeh A, Flint-Ofir E, Tirosh T, Tikotzky L (2007). Infant sleep and parental sleep-related cognitions. J Fam Psychol.

[77] Sadeh A (1994). Assessment of intervention for infant night waking: parental reports and activity-based home monitoring. J Consult Clin Psychol.

[78] Belza BL (1995). Comparison of self-reported fatigue in rheumatoid arthritis and controls. J Rheumatol.

[79] Salmon P, Drew NC (1992). Multidimensional assessment of women’s experience of childbirth: relationship to obstetric procedure, antenatal preparation and obstetric history. J Psychosom Res.

[80] Dennis C, Heaman M, Mossman M (2011). Psychometric testing of the breastfeeding self-efficacy scale-short form among adolescents. J Adolesc Health.

[81] de Bruin EI, Topper M, Muskens JG, Bögels SM, Kamphuis JH (2012). Psychometric properties of the Five Facets Mindfulness Questionnaire (FFMQ) in a meditating and a non-meditating sample. Assessment.

[82] de Bruin EI, Zijlstra BJ, Geurtzen N, van Zundert RM, van de Weijer-Bergsma E, Hartman EE, Pouwer F, Duncan LG, Bögels SM (2013). Mindful parenting assessed further: Psychometric properties of the Dutch version of the Interpersonal Mindfulness in Parenting Scale (IM-P). Mindfulness.

[83] Neff KD (2003). Development and validation of a scale to measure self-compassion. Self Identity.

[84] Tan SS, Bouwmans CA, Rutten FF, Hakkaart-van RL (2012). Update of the Dutch manual for costing in economic evaluations. Int J Technol Assess Health Care.

[85] Briggs AH, Wonderling DE, Mooney CZ (1997). Pulling cost-effectiveness analysis up by its bootstraps: a non-parametric approach to confidence interval estimation. Health Econ.

[86] Higgins JPT, Green S. Cochrane Handbook for Systematic Reviews of Interventions. Version 5.1.0. Part 3, Chapter 13. 2011. http://handbook.cochrane.org/whnjs.htm. Accessed 20 Mar 2011.

